# Molecular heterogeneity of guanine nucleotide binding-protein γ subunit 4 in left- and right-sided colon cancer

**DOI:** 10.3892/ol.2022.13597

**Published:** 2022-11-16

**Authors:** Jintian Song, Jianwei Yang, Rongbo Lin, Xiongchao Cai, Liang Zheng, Yigui Chen

Oncol Lett 20: 334, 2020; DOI: 10.3892/ol.2020.12197

Subsequently to the publication of the above article, and a previously published corrigendum that addressed the issue of an incorrect author affiliation (DOI: 10.3892/ol.2021.12488; published online, January 26 2021), the authors have realized that Fig. 4C on p. 6 was published with an incorrectly selected image; specifically, the wrong data were chosen for the high-power ‘Left-sided colon cancer’ immunohistochemistry (IHC) image.

The revised version of Fig. 4, showing the correct data for the high-power ‘Left-sided colon cancer’ IHC image in Fig 4C, is shown on the next page. Note that the error made in this Figure did not affect the results or the conclusions reported in this paper, and all the authors agree to this Corrigendum. The authors thank the Editor of *Oncology Letters* for presenting them with the opportunity to publish this Corrigendum, and apologize to the Editor and to the readership of the Journal for any inconvenience caused.

## Figures and Tables

**Figure 4.**
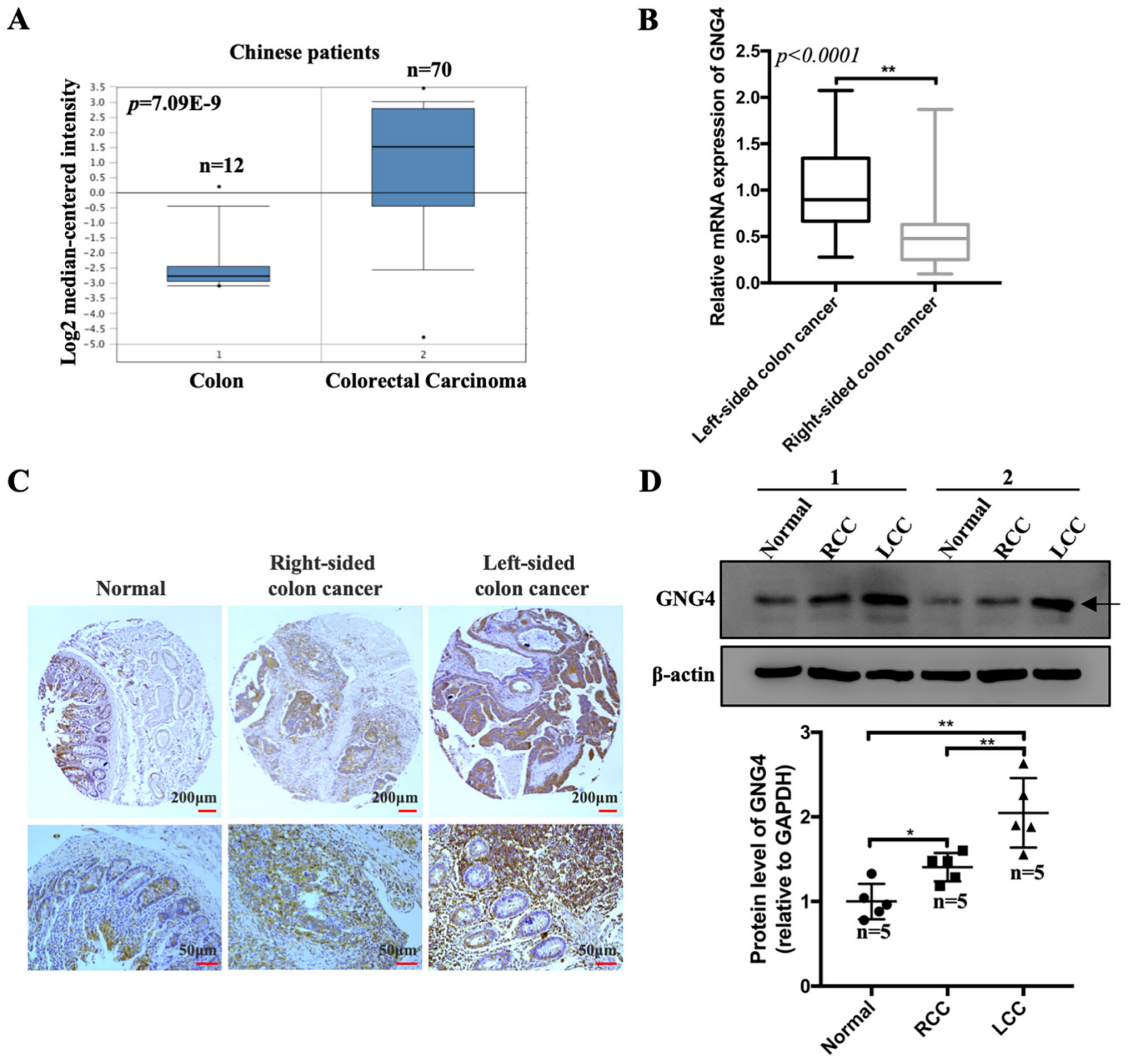
Differential analysis of GNG4 expression in Chinese patients with COAD. (A) Transcriptional expression levels of GNG4 in normal controls and patients with COAD. The raw data were obtained from the Oncomine database. Data were analyzed with GraphPad Prism using two-tailed t-tests (unpaired). (B) Differential GNG4 mRNA expression between LCC and RCC was determined using reverse transcription-quantitative PCR. (C) Immunohistochemical staining of GNG4 in normal, LCC and RCC tissues in the tissue microarray. (D) Protein expression levels of GNG4 in normal, LCC and RCC tissues, as measured by western blotting. The graph presents the relative protein expression levels of GNG4 in the aforementioned three tissues (one-way ANOVA, followed by Tukey's post hoc test). *P<0.05; **P<0.01. COAD, colon adenocarcinoma; GNG4, guanine nucleotide binding-protein γ subunit 4; LCC, left-sided colon cancer; RCC, right=sided colon cancer.

